# Quantifying Use of a Health Virtual Community of Practice for General Practitioners’ Continuing Professional Development: A Novel Methodology and Pilot Evaluation

**DOI:** 10.2196/14545

**Published:** 2019-11-27

**Authors:** Abdulaziz Murad, Natalie Hyde, Shanton Chang, Reeva Lederman, Rachelle Bosua, Marie Pirotta, Ralph Audehm, Christopher J Yates, Andrew M Briggs, Alexandra Gorelik, Cherie Chiang, John D Wark

**Affiliations:** 1 School of Computing and Information Systems University of Melbourne Melbourne Australia; 2 Deakin University Geelong Australia; 3 Open University of the Netherlands Heerlen Netherlands; 4 Department of General Practice University of Melbourne Melbourne Australia; 5 Department of Medicine Royal Melbourne Hospital University of Melbourne Melbourne Australia; 6 Department of Diabetes and Endocrinology Royal Melbourne Hospital Melbourne Australia; 7 Bone and Mineral Medicine Royal Melbourne Hospital Melbourne Australia; 8 School of Physiotherapy and Exercise Science Faculty of Health Sciences Curtin University Perth Australia; 9 School of Behavioral and Health Science Australian Catholic University Melbourne Australia; 10 Department of Pathology Royal Melbourne Hospital Melbourne Australia

**Keywords:** online systems, online social networking, general practitioners, online learning, continuing education, professional education, evaluation methodology, use-effectiveness, quantitative evaluation, knowledgebases, information sharing

## Abstract

**Background:**

Health care practitioners (HPs), in particular general practitioners (GPs), are increasingly adopting Web-based social media platforms for continuing professional development (CPD). As GPs are restricted by time, distance, and demanding workloads, a health virtual community of practice (HVCoP) is an ideal solution to replace face-to-face CPD with Web-based CPD. However, barriers such as time and work schedules may limit participation in an HVCoP. Furthermore, it is difficult to gauge whether GPs engage actively or passively in HVCoP knowledge-acquisition for Web-based CPD, as GPs’ competencies are usually measured with pre- and posttests.

**Objective:**

This study investigated a method for measuring the engagement features needed for an HVCoP (the Community Fracture Capture [CFC] Learning Hub) for learning and knowledge sharing among GPs for their CPD activity.

**Methods:**

A prototype CFC Learning Hub was developed using an Igloo Web-based social media software platform and involved a convenience sample of GPs interested in bone health topics. This Hub, a secure Web-based community site, included 2 key components: an online discussion forum and a knowledge repository (the Knowledge Hub). The discussion forum contained anonymized case studies (contributed by GP participants) and topical discussions (topics that were not case studies). Using 2 complementary tools (Google Analytics and Igloo Statistical Tool), we characterized individual participating GPs’ engagement with the Hub. We measured the GP participants’ behavior by quantifying the number of online sessions of the participants, activities undertaken within these online sessions, written posts made per learning topic, and their time spent per topic. We calculated time spent in both active and passive engagement for each topic.

**Results:**

Seven GPs participated in the CFC Learning Hub HVCoP from September to November 2017. The complementary tools successfully captured the GP participants’ engagement in the Hub. GPs were more active in topics in the discussion forum that had direct clinical application as opposed to didactic, evidence-based discussion topics (ie, topical discussions). From our knowledge hub, About Osteoporosis and Prevention were the most engaging topics, whereas shared decision making was the least active topic.

**Conclusions:**

We showcased a novel complementary analysis method that allowed us to quantify the CFC Learning Hub’s usage data into (1) sessions, (2) activities, (3) active or passive time spent, and (4) posts made to evaluate the potential engagement features needed for an HVCoP focused on GP participants’ CPD process. Our design and evaluation methods for ongoing use and engagement in this Hub may be useful to evaluate future learning and knowledge-sharing projects for GPs and may allow for extension to other HPs’ environments. However, owing to the limited number of GP participants in this study, we suggest that further research with a larger cohort should be performed to validate and extend these findings.

## Introduction

### Background

Knowledgeable and skillful general practitioners (GPs) are fundamental for efficient and effective health care systems. In most communities, they are the primary source of health care to individuals and families [1,2], lending them the name *family practitioner/physician* [2,3]. Owing to the important role of GPs in the community, high-quality training and continuing professional development (CPD) are of utmost importance [2], creating an imperative to provide the necessary learning that GPs require to remain competent in their field [4,5]. GPs are required to continuously expand their knowledge and skills to ensure that evidence-based research is applied to provide the best care possible to patients [4,6]. Health care practitioners (HPs), GPs in particular, can find it challenging to maintain their CPD activities considering their heavy and often demanding workloads. Moreover, GPs and other HPs who practice in relatively isolated regions, which are common in a country such as Australia, can find it particularly difficult to participate in traditional educational activities. Therefore, new ways are required to enable and support efforts to maintain CPD activities.

GPs have become accustomed to using networking events (eg, conferences and out-of-hour lectures) and extensive reading to acquire new knowledge [7]. In addition, GPs seem to appreciate Web-based information to further develop practices and are increasingly seeking information online for their CPD [5,8-10]. GPs understand that Web-based CPD is a new, viable alternative to face-to-face learning (ie, conferences) that can be managed in their own time to further develop their learning and knowledge-sharing competencies for practice [11]. Hence, incorporating social media technologies for CPD has become a commonplace mechanism encouraging GPs’ learning in online group settings (eg, Facebook and Twitter) [2,5,8].

A practitioner group working together on shared practices is defined as a community of practice. Health virtual communities of practice (HVCoPs) refer to a class of internet technologies used to share the best practices among HPs [8,12]. As a result of HVCoPs, Web-based community-based learning, sharing, and adopting of explicit evidence-based medical knowledge in work practices [8] by GPs [13] have arisen in the past decade.

Although HPs/GPs recognize the potential of using social media technologies for learning and knowledge sharing [8], they question whether using Web-based communities (eg, Facebook) to gain knowledge and share experiences for CPD is acceptable owing to privacy and trust issues [14,15]. Patient information being identifiable (eg, a rare disease that only a handful of patients have) to other online participants was considered a privacy concern. Another known privacy issue was GPs’ personal information being identifiable (eg, identifying GPs by their location of practice) with constant online activity of sharing information. Not knowing if participants are real, practicing GPs or a random person online impersonating a GP was considered a trust issue. These concerns have lowered engagement over time [16]. GPs have also found it difficult to use other tools (eg, Web-based databases) to search for evidence-based research results, resulting in users and facilitators abandoning HVCoP systems [17]. Some GPs have also experienced difficulties in using mobile apps specifically developed to support literature searches [18]. GPs also differ in the type of CPD they require depending on the terms of appraisal needed or whether they are seeking to review existing knowledge or gain new knowledge [19]. Furthermore, GPs tend to lose interest over time even when fully engaged in an HVCoP (ie, user participation decreases after 2-3 months) as engagement for learning and knowledge-sharing activities in online forums is demanding on the participants’ involvement (ie, users and facilitators) [13,20]. GPs often face other demands on their time that may limit participation [8,13]. As such, Web-based learning has not yet proven its benefits for learning, knowledge acquisition, and cost-effectiveness for GPs using Web-based systems [21]. Previous work (Multimedia Appendix 1) supported a set of required design principles to evaluate GPs’ engagement through participation in our Community Fracture Capture (CFC) Learning Hub (an interactive, case-based, Web-based learning tool designed to help GPs improve the care of patients in relation to osteoporosis). It was hoped that such an HVCoP would help to mitigate barriers to participation in Web-based learning [22-24]. Furthermore, active and passive GP engagement has not been formally studied in HVCoPs, as there has been no real way to track and quantify the participants’ usage behavior. *Active users* post constantly in online discussion forums, whereas passive users merely engage in viewing content with no posting activity [13]. Previous studies typically [8,13,17,25] employed pre- and posttests to gauge whether GP participants, active or passive, acquired knowledge from the HVCoP to measure user engagement. Pre- and posttest evaluations for Web-based CPD are the most common forms of assessment, and there is a need for additional evaluation methods for GPs’ learning outcomes [21]. Moreover, we are not aware of any previously described methods to measure GPs’ engagement behaviors (ie, activity/usage) with Web-based learning CPD platforms at the individual level, let alone in the HVCoPs. Hence, to our knowledge, there are no current HVCoP platforms or studies that measure active and passive behavior to gauge GP participants’ engagement.

### Objectives

There is a need to better quantify GPs’ participation in CPD through HVCoP usage, to establish whether active or passively engaged GPs’ truly learn and acquire knowledge. In this paper, we report preliminary findings of GPs’ learning and knowledge sharing in an HVCoP for CPD. In this ongoing research program to design and evaluate an HVCoP for GPs’ CPD endeavors [22-24], we pose the research question: *What engagement features promote the use of an HVCoP for GPs’ CPD?* This paper aimed to present the findings of a project undertaken to describe the performance features of a customized, interactive, case-based learning hub designed to help improve GPs’ understanding and management of osteoporosis, a common condition that remains underdiagnosed and undertreated in many countries [26]. Hence, the main purpose of this study was the development and demonstration of a novel design and methodology for CPD and its evaluation.

## Methods

### The Prototype Platform

The CFC Learning Hub is a secure, Web-based prototype HVCoP website, created using the *Igloo* Web-based social media software platform. The CFC Learning Hub was developed for enhancing GPs’ awareness and competence in caring for patients with osteoporosis. The project team comprised a mix of specialists in the field: experienced GPs; information systems researchers; technology experts; a project coordinator; and specialist physicians with expertise in bone health and osteoporosis, the CFC Learning Hub’s theme. The project team, situated in Melbourne, Australia, drew on its learning, teaching, and clinical experience to define the following important design criteria for GP participants and elements in the project:

Practicing GPs was the target group, with potential inclusion of practice nurses (though there was some uncertainty whether this might inhibit contribution by some GPs).

Case-based learning preferably involving both experienced and trainee GPs’ own case study contributions with anonymized patients (to encourage engagement).

Interactive engagement among all parties involved, that is, no didactic teaching component.

GP peers led the group as facilitators, with guidance where needed by specialist *advisers*, promoting case discussions and guiding discussion of content according to relevance and importance.

The total time commitment required for the CPD trial to make effective use of GPs’ limited time, whereas the CFC Learning Hub format was to be flexible in terms of the timing of GP participants’ input in the form of contributions (to more easily fit in with their workload commitments).

The CFC Learning Hub platform was developed with the assistance of an external developing entity (Involved—Design and Development Agency, Melbourne, Australia), which agreed that Igloo Web-based social media software was the best platform, kept development costs moderate, and accommodated the investigation team’s design criteria. Our project team worked closely with Involved to develop and implement the CFC Learning Hub prototype with the support of 3 external GPs in a collaborative approach to the design of the HVCoP.

A facilitation team, formed to incentivize the engagement of the participants and facilitate discussions in the online discussion forum, included 4 specialist physicians, 2 senior GPs as facilitators, 1 dedicated content facilitator, and 1 information technology administrator. This team had moderation and administration rights throughout the CFC Learning Hub’s life, whereas GP participants who joined and contributed case studies remained as participants throughout this period. GP participants were deidentified with an anonymous key for data management and analysis.

The CFC Learning Hub platform had 2 resources for GP interactions:

1. A Web-based knowledge repository (the Knowledge Hub) containing curated and prepopulated evidence-based research articles and other resources.
2. An online social network forum (the *Discussion Forum*) where GPs could freely post online comments, including:
  - Questions for discussion posted by facilitators;
  - Case studies to encourage GPs to learn and share their knowledge based on shared experiences and relevance to their immediate clinical practice;
  - Topical discussions as either (a) hot topics (HT) deemed relevant for GPs, posted by our osteoporosis specialists or (b) other topics (OT) that were open for wider discussion (ie, introductions, where facilitators and GPs introduce themselves, and burning questions, where GPs and facilitators post inquiries on osteoporosis).

The GP participants provided case studies as a requirement for joining the CFC Learning Hub. Facilitators and specialist advisers filtered and chose case studies that enabled the coverage of a syllabus of topics predetermined by members of the project team for this CPD course. Facilitators ensured that the posted case studies were anonymized and contained suitable, high-quality content for discussion. A total of 6 chosen case studies were posted for discussion in the CFC Learning Hub at approximately weekly intervals.

The platform’s topical discussions section included all OT posted in the discussion forum of the CFC Learning Hub. These included (1) an HT section chosen by the facilitators and specialist advisers to enrich GP participants’ learning and knowledge sharing on issues identified as being of particular importance: diabetes and bone health, atypical femoral fracture, when to consider changing an osteoporosis therapy, and how to get the most from your patients’ bone density testing; (2) an *Introduction* topic for all users to introduce themselves; and (3) a facility for GP participants to post inquiries based on seeking specific information about osteoporosis. Facilitators also could raise questions to promote discussion. These inquiries were organized under the term, *Burning Questions*. In terms of privacy, the CFC Learning Hub itself was a private network with a password log-in functionality that excluded online public entities (ie, people or organizations) outside of the HVCoP. In terms of trust, all GP participants and facilitators were known to each other within the CFC Learning Hub as all had customizable profiles and were not anonymized. We adopted this approach to instill trust among the participants as being genuine participants, consistent with standard Web-based private learning environments.

The knowledge hub acted as an accessible knowledge repository for all users at all times. This hub had 7 topics that the project team chose to include, each having detailed evidence-based research articles online. Furthermore, any new and interesting topics that the GP participants were discussing in the online discussion forum could be added at a later time by the facilitator team.

The lead time to the commencement of the CFC Learning Hub was 2.2 years (from December 2014 to February 2017). On average, the project lead (JDW) spent approximately 3 hours per week during the lead time to the project launch and 1.5 hours per week during the active phase of the project. The time spent by the investigation team was an average of 1 hour per project team member per fortnight leading to the launch of the CFC Learning Hub, and an average of 0.5 to 1 hour per week during the active phase of the project. The information systems doctoral student (AM) spent approximately 1 hour per week during the lead time to the project launch, and 3.5 hours per week during the active phase of the project (ie, tracking live interactions to update facilitators on engagement). The project coordinator (NH) was appointed at 0.4 full time equivalent during the lead time of the project. The developers (Involved—Design and Development Agency) had 5 meetings with the project team and proposed a work timeline to develop the CFC Learning Hub in 2 days, with time to be split between a producer, developer, and designer.

The direct project costs were Aus $170,000 (approximately US $114,000) and covered the following: Web developer fees, database management, Web hosting, content development, Igloo platform fees, ethics and governance submission fees, part-time study coordinator salary, GP facilitator consultancies, statistics support, and anticipated publication costs. In addition, AM received full-time support by a doctoral scholarship.

Upon the launch of the CFC Learning Hub, the time spent by facilitators depended on each facilitator, but there was an agreed expectation to be available once per day. Specialists involved from the facilitation team scheduled themselves with the assistance of the project coordinator, for each to be dedicated for a specific week of the CFC Learning Hub’s full life cycle.

**Figure 1:**
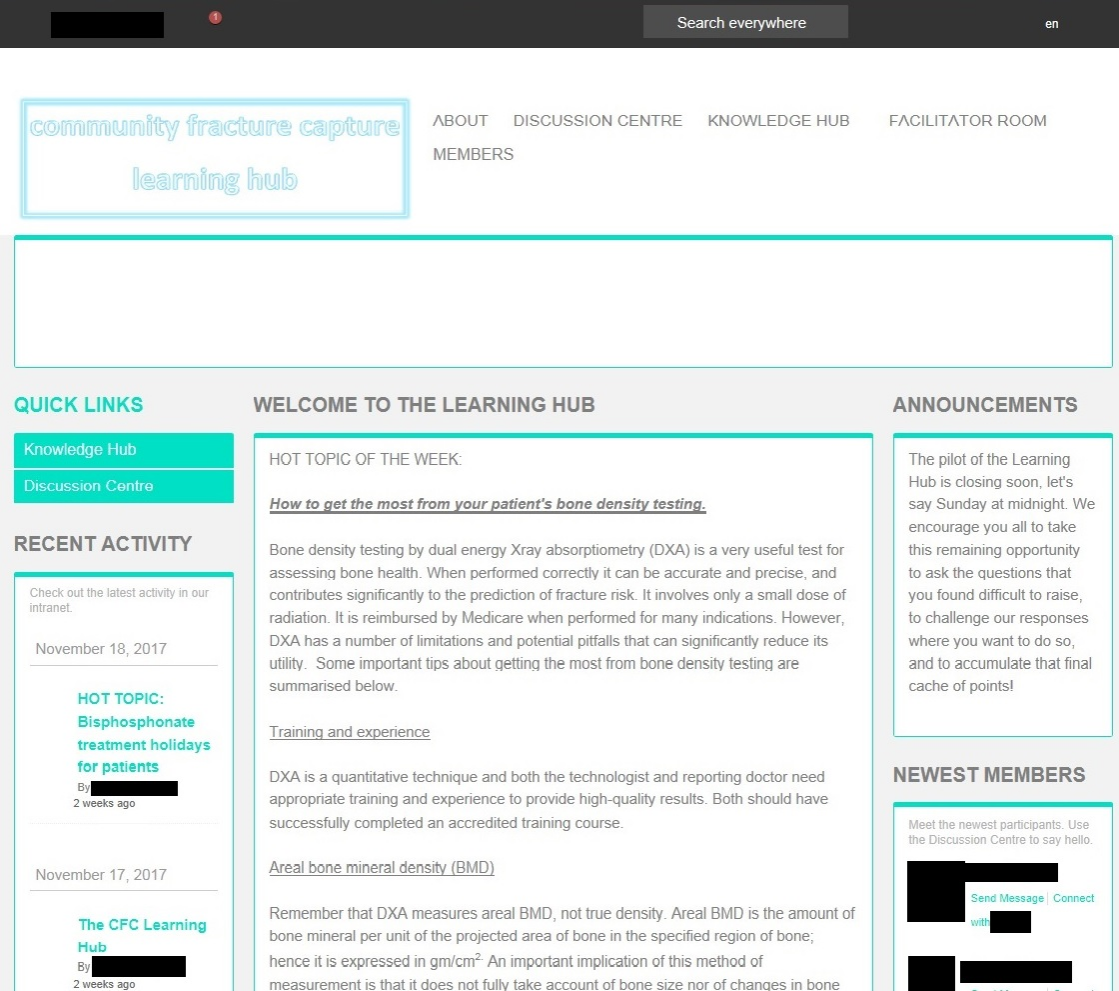
The Community Fracture Capture Learning Hub home screen.

**Figure 2:**
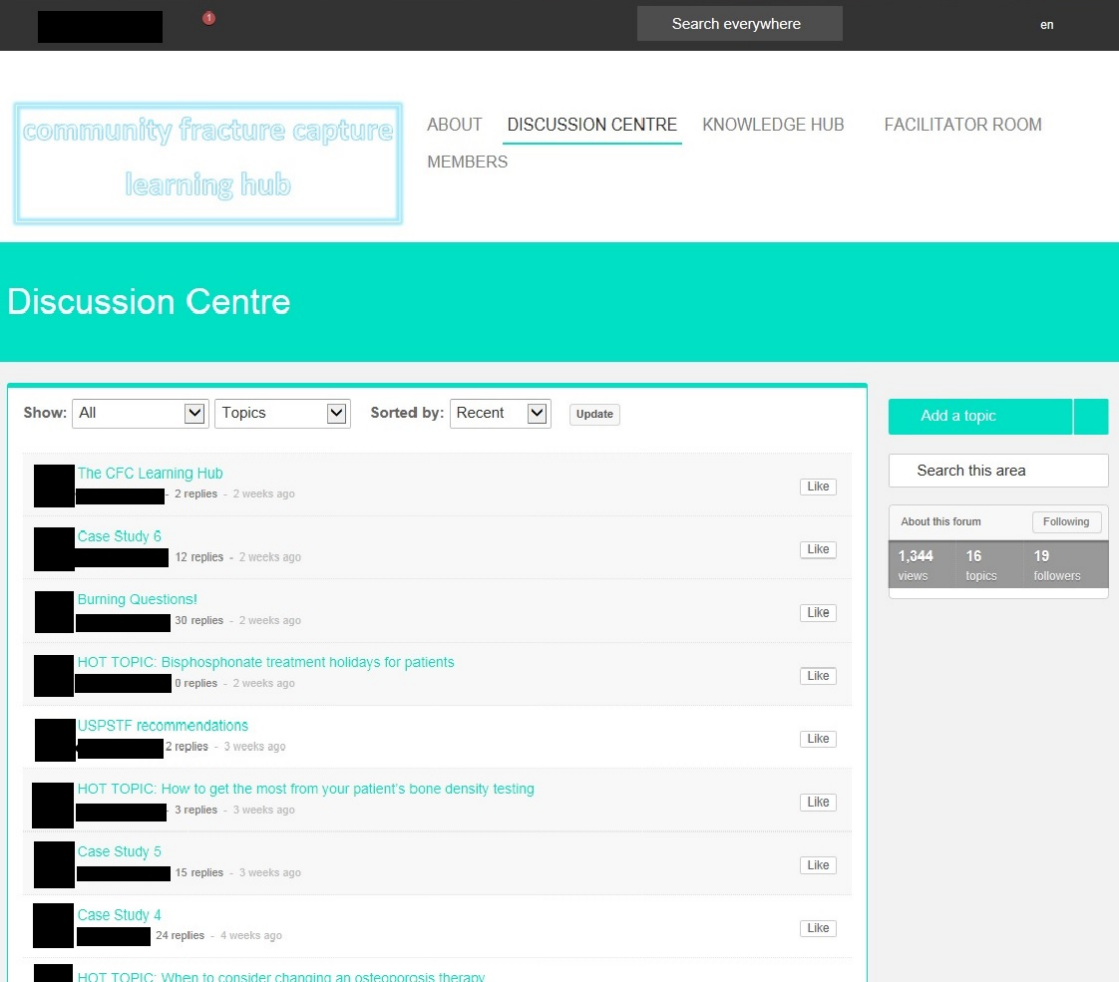
A brief graphical representation of using 2 analytical tools to process data.

Figure 1 is a screenshot of the home screen and Figure 2 is an example of its discussion forum.

### Study Site

The study site was managed at the University of Melbourne and the Royal Melbourne Hospital. All users could sign up for password access to the CFC Learning Hub from one or more sites, for example, home or practice.

### Data Storage and Security

All knowledge repository content was stored electronically on the Igloo data center platform. Each user had his/her own unique username and password to access the CFC Learning Hub. User data were also held in the Igloo data center in Toronto and Vancouver, Canada. GP participants were assigned a unique study number, and all collected data were deidentified (anonymized) and made reidentifiable only by linking separately stored password-protected GP participant information. All study data were password protected and accessible only with the approval of the study’s principal investigator.

### Study Timeline

#### Recruitment

The recruitment period was from February until August 2017.

#### Participation in Online Community Fracture Capture Learning Hub

The Web-based CPD course proceeded over 8 weeks (September to November 2017). Users received a 2-week introductory/familiarization period at the beginning of the course. Following this, the 6-week active period of the CFC Learning Hub commenced.

### Study Population

#### Recruitment Procedure

The recruitment target was 15 GPs, identified from the GP participants of an earlier industry-sponsored osteoporosis education program and the Victorian Primary Care Practice-Based Research Network (VicReN) [27] mailing list. These GPs were sent a flyer inviting their participation in the project. Interested GPs contacted the project coordinator, after which they were screened for eligibility to participate in the program and subsequently enrolled if they satisfied the selection criteria and consented to participate.

#### Inclusion Criteria

GP participants were required to be a medical practitioner currently active in general practice in Australia, to submit preferably 2 or at least one suitable case study with discussion or learning points from their own clinical experience that included patients at risk or those with prevalent osteoporosis, and to have internet access.

#### Exclusion Criteria

GP participants excluded were those who were unable to provide patient case studies for discussion or were unable to commit to the anticipated time required to meet appropriate CPD guidelines (at least six hours).

### Measures

Data related to sessions and activities of GP participants in the CFC Learning Hub were collected for all 8 weeks. Our focus was on the usage data to identify the GPs’ learning and knowledge-sharing behaviors related to participation and engagement in the CFC Learning Hub. We defined the following terms from Google Analytics, henceforth termed GA:

- A session included all user-related activities from initial logging in to logging out of the CFC Learning Hub. All activities made between the logging in and logging out activities were grouped within a session.
- An activity included downloading of content, viewing posts and content, and posting comments.

The measurement of sessions and activities of a GP participant covered his or her entire active and passive engagement with the Web-based system, which could be used to measure the success of the prototype platform system (ie, high session count meant GPs were logging in, undertaking one or more activities, and then logging out). Actively engaged GPs were GP participants who posted a case study/topical discussion, whereas passively engaged GPs were GP participants who did not post but spent time only browsing a case study/topical discussion. In the knowledge hub, all GPs’ participation was inherently passive as GPs were all browsing the knowledge hub database.

For measuring the use of the discussion forum, the usage behavior for each case study and topical discussion was assessed. For each case study and topical discussion, usage was measured from the creation date until the end of a 7-day period commencing from the creation date.

The use of the discussion forum was measured for the full CPD trial period (2 months) looking at the following:

- Each case study/topical discussion and its related discussion sessions for GP participants during the week after the case study/topical discussion was first published online. This showed the distribution of sessions among case studies/topical discussions and whether the GPs returned seeking more information, learning, and discussion. In addition, we assessed case studies/topical discussions that attracted the most activity by GPs.
- How long GP participants spent on each case study/topical discussion each week.
- The number of posts each GP made per case study/topical discussion per week. This measure also showed GPs who were actively and passively engaged per case study/topical discussion.

For the knowledge hub, we followed 2 key points in identifying engaging topics for GPs’ CPD from a learning and knowledge-sharing perspective: (1) examining each knowledge hub’s unique GP participant sessions and activities and (2) calculating how long GP participants spent on each knowledge hub topic.

### Data Collection

Data were collected from 2 main sources: (1) the Igloo statistical tool, henceforth termed IGT, which can track the user’s identification (eg, user 1 is John Doe) and (2) GA, which captures cumulative real-time use by users. However, in GA, users are not identifiable.

Both IGT and GA were used to collect and analyze the time that GP participants spent on each topic in the discussion forum and knowledge hub as follows:

- GA captured all users’ (including facilitators and GP participants) sessions and activities in the CFC Learning Hub. However, all users were anonymous by default owing to GA’s privacy terms and conditions. Hence, IGT was used to verify each activity, as IGT correctly identified each user ID in the CFC Learning Hub. This process was conducted manually to collate sessions and activities and avoid errors in identifying GP participants.
- IGT does not track how long each user spent on a given activity. However, GA tracked all activities and their duration for each user ID with time stamps. Hence, the 2 sets of results were cross-referenced with the time spent being calculated manually.

Although GA captures all facilitators’ and GP participants’ activities, GA does not differentiate between a view, download, or comment for each user, as it merges these together into 1 *Viewing* heading. IGT solves this issue by indicating each user’s activity in detail but grouped together on a given period (eg, GP participant commented thrice on a given day). Hence, GA was relied upon to capture the activity of each GP participant, complemented by IGT to determine the type of activity for each individual, followed by cross-referencing with GA for the duration of activities. GA captured the cumulative real-time use of the participants. If a participant stopped using the platform for more than 30 min (the user opened the website page and became idle, ie, was not moving the mouse or was looking at another Web page), GA would automatically not count a session.

This method also measured whether GP participants were active (ie, posting comments to discuss) or passive (ie, viewing only). All data were collated in Microsoft Excel. See Multimedia Appendix 2.

### Ethical Approval

The Melbourne Health Human Research Ethics Committee approved this project (site reference number: 2016.24). Electronic written consent from GP participants who joined the CFC Learning Hub was obtained to use their data. Electronic written consent data were collected and managed by using Research Electronic Data Capture (REDCap) tools hosted at the University of Melbourne [28]. REDCap is a secure, Web-based application designed to support data capture for research studies, providing (1) an intuitive interface for validated data entry, (2) audit trails for tracking data manipulation and export procedures, (3) automated export procedures for seamless data downloads to common statistical packages, and (4) procedures for importing data from external sources [28]. The electronic consent also included GP waiver of consent of case study patients whose anonymized information was used in the project.

## Results

### Recruitment

A total of 19 GPs showed initial interest in joining the CFC Learning Hub. Sources of recruitment included VicReN, personal contacts from our GP facilitators, and from the Australian College of Rural and Remote Medicine website’s CPD offerings. Of these 19 GPs, 8 committed to join. Later, 1 GP participant dropped out owing to family reasons, and 7 GPs continued for the full 8-week CPD course duration. Overall, 3 GP participants were above 50 years of age and 4 GP participants were aged under 50 years. Furthermore, 6 GP participants were females, and 1 was male. A total of 5 GP participants had more than 5 years of practice experience and 2 GP participants had less than 5 years of practice experience.

### Case Studies

Using IGT and GA’s complementary tools to collect and process data, we were able to identify, from over 90 unique IDs, 7 GP participants’ use of various technologies (mobile, desktop, or tablet, given by GA specifically).

The case studies are described briefly in Table 1. In addition, this section highlights GP users’ engagement in the case studies discussed in the discussion forum.

The total number of case study session engagement for all GP participants during their first week of creation:

- Case study 1 had 16.
- Case study 2 had 15.
- Case study 3 had 26.
- Case study 4 had 25.
- Case study 5 had 26.
- Case study 6 had 19.

The total number of sessions by all GP participants per case study topic ranged from 15 to 26, and the median number of GP participant sessions per topic was 22. We recorded 127 GP participant sessions in total.

The total number of topical discussion activities engagement for all GP participants during their first week of creation:

- Case study 1 had 17.
- Case study 2 had 20.
- Case study 3 had 31.
- Case study 4 had 31.
- Case study 5 had 32.
- Case study 6 had 26.

The total number of activities by all GP participants ranged from 17 to 32, and the median number of activities per topic was 28.5. In total there were 148 activities.

Table 2 presents the time spent by GP participants for each case study and the number of posts made for each given week, actively and passively. Table 2 presents a description of the engagement in each case study.

From Table 2, the total time spent per case study by all GP participants ranged from 32 min to 114 min, with a median of 86.5 min per case study. All GP participants put together spent a total of 458 min for all case studies.

The number of posts per case study ranged from 3 to 8, and the median number of GP participant posts was 5.5 for all case studies. The median number of active GP participants was 4 and the median number of passive GP participants was 2, for all case studies.

**Table 1:** Description of each case study’s content.

Case studies	Description of case study content
Case study 1	A 59-year-old woman having bone mineral density measured as a health check
Case study 2	A 56-year-old woman with osteoporosis managed with raloxifene and physical activity
Case study 3	A 70-year-old woman with several osteoporosis risk factors
Case study 4	Osteoporosis in a 70-year-old man on androgen deprivation therapy postprostatectomy for prostate cancer
Case study 5	An 89-year-old woman with a history of vertebral fracture and previous osteoporosis therapy
Case study 6	A 67-year-old woman who has lost height and has dental problems

**Table 2:** Time spent (minutes) and posts made by all general practitioner participants on each case study during the first week of its creation. Active, passive, and nonengagements are included.

Case studies	Time spent in sessions by all GP^a^ participants (min)	Posts made by all GP participants, n	GP participants actively engaged^b^, n	GP participants passively engaged^c^, n	GP participants not engaged, n
Case study 1	32	8	2	2	3
Case study 2	81	4	3	2	2
Case study 3	93	3	2	3	2
Case study 4	92	7	5	2	0
Case study 5	114	7	5	0	2
Case study 6	46	4	4	2	1
Total	458	33	N/A^d^	N/A	N/A

^a^GP: general practitioner.

^b^Participants who posted, browsed, and downloaded content.

^c^Participants who did not post but spent time browsing and downloading content.

^d^N/A: not applicable.

### Topical Discussions

This section highlights the GP participants’ engagement in topical discussions on the discussion forum of the CFC Learning Hub.

The total number of topical discussion session engagement for all GP participants during their first week of creation:

- Other topics 1 (OT1) Introduction had 3.
- Hot topic 1 (HT1): Diabetes and bone health had 8.
- Hot topic 2 (HT2): Atypical femoral fracture had 9
- Hot topic 3 (HT3): When to consider changing an osteoporosis therapy had 11.
- Hot topic 4 (HT4): How to get the most from your patients bone density testing had 6.
- Other topics 2 (OT2): Burning questions had 18.

The total number of sessions per topical discussion ranged from 3 to 18, and the median number of GP participant sessions per topical discussion was 8.5. The total number of sessions made by GP participants for all topical discussion was 55.

The total number of topical discussion activities engagement for all GP participants during their first week of creation:

- Other topics 1 (OT1) Introduction had 3.
- Hot topic 1 (HT1): Diabetes and bone health had 9.
- Hot topic 2 (HT2): Atypical femoral fracture had 9.
- Hot topic 3 (HT3): When to consider changing an osteoporosis therapy had 12.
- Hot topic 4 (HT4): How to get the most from your patients bone density testing had 7.
- Other topics 2 (OT2): Burning questions had 22.

The total number of activities per topical discussion ranged from 3 to 22, and the median per topic was 9. The total number of GP participant activities for all topical discussions was 57.

Table 3 presents the time spent by the GP participants on each topical discussion and the number of posts made for each given week, actively and passively.

In total, there were 14 burning questions covering 9 topics. The questions came from participating GPs and from GP facilitators. The topics were the assessment of an older patient with a recent peripheral fracture, the management of bone health in a young woman with an eating disorder, the selection of osteoporosis therapy, when to refer an osteoporosis patient to a specialist, the risks with osteoporosis therapy, what could GPs do better for their osteoporosis patients, the management of bone health in wheelchair-bound patients, the use of denosumab in patients with renal impairment, and the choice of therapy following a course of teriparatide.

As shown in Table 3, the total time spent by GP participants per topical discussion session ranged from 3 min to 72 min. The median time spent by all GPs for topical discussions was 19 min. GP participants spent a cumulative time of 189 min on topical discussions.

The post count by GP participants per topical discussion ranged from 0 to 10, and the median post count for all GP participants was 2. The total posts by GP participants for all topical discussions were 16. However, it should be noted that there were 14 burning questions; therefore, the number of posts for burning questions cannot be directly compared with those for HT. The median number of actively engaged GP participants was 1 and the median number of passively engaged GPs was 3, for all topical discussions.

**Table 3:** Total time spent (minutes) and posts made by general practitioner participants on each topical discussion during the first week of its creation. Active, passive, and nonengagements are included.

Topical discussions	Time spent in sessions by all GP^a^ participants (min)	Posts made by all GP participants, n	GP participants actively engaged, n	GP participants passively engaged, n	GP participants not engaged, n
Other topic 1: Introductions	3	0	0	3	4
Hot topic 1: Diabetes and bone health	10	1	1	3	3
Hot topic 2: Atypical femoral fracture	61	3	2	2	3
Hot topic 3: When to consider changing an osteoporosis therapy	24	1	1	4	2
Hot topic 4: How to get the most from your patient’s bone density testing	19	1	1	4	2
Other topic 2: Burning questions^b^	72	10	2	2	3
Total	189	16	NA^c^	NA	NA

^a^GP: general practitioner.

^b^This category contained 14 questions and 9 topics.

^c^N/A: Not applicable.

### Knowledge Hub

This section includes GP participants who were engaged for sessions and activities in the knowledge hub from the CFC Learning Hub.

The total number of Knowledge Hub sessions engagement by all GP participants throughout the trial:

- KH1: About osteoporosis had 10.
- KH2: Diagnosis had 5.
- KH3: Patient resources had 6.
- KH4: Prevention had 8.
- KH5: Risk assessment had 10.
- KH6: Shared decision making had 0.
- KH7: Treatment had 9.

The number of sessions per knowledge hub topic ranged from 0 to 10, and the median per knowledge hub topic by all GP participants was 8. In total, GP participants undertook 48 knowledge hub sessions.

The total number of Knowledge Hub activities engagement by all GP participants throughout the trial:

- KH1: About osteoporosis had 12.
- KH2: Diagnosis had 6.
- KH3: Patient resources had 9.
- KH4: Prevention had 9.
- KH5: Risk assessment had 14.
- KH6: Shared decision making had 0.
- KH7: Treatment had 14.

The number of GP participants’ activities per knowledge hub topic ranged from 0 to 14, with a total of 64 activities and a median of 9 activities per knowledge hub topic.

Table 4 presents the total time spent on each knowledge hub topic in sessions by all GP participants and the number of GP participants who engaged in each knowledge hub topic over the duration of the trial.

As shown in Table 4, the time spent by GP participants in the knowledge hub ranged from 0 min to 226 min per knowledge hub topic, with the median time spent being 152 min. The total time spent by GP participants was 1057 min. The number of GP participants who engaged in each topic ranged from 0 to 6. The median number who engaged per topic was 4 and the median number of nonengaged participants per topic was 3.

**Table 4:** Time spent (minutes) by general practitioner participants in sessions and the number who engaged in each knowledge hub topic.

KH^a^ topic	Total time spent by all GP^b^ participants on KH topics (min)	GP participants engaged in each KH topic^c^, n	GP participants not engaged in each KH topic, n
KH1: About osteoporosis	223	6	1
KH2: Diagnosis	143	4	3
KH3: Patient resources	140	3	4
KH4: Prevention	226	6	1
KH5: Risk assessment	152	4	3
KH6: Shared decision making	0	0	7
KH7: Treatment	173	4	3
Total	1057	N/A^d^	N/A

^a^KH: knowledge hub.

^b^GP: general practitioner.

^c^Engagement in knowledge hub topics was passive only.

^d^N/A: not applicable.

## Discussion

### Principal Findings

The objective of this study was to test the methods we developed to quantify GP participants’ active and passive interactions within an HVCoP platform, which we designed as a learning tool for patient care in osteoporosis. Furthermore, to our knowledge no other HVCoP reported in the literature had the ability to capture individual GPs’ engagement in detail, which our study managed to do, and to explain the intricacies of the method itself. The key features of the platform were that the material presented was centered around the clinical cases provided by the participating GPs themselves and that the learning activities were designed to be interactive. In this functional HVCoP platform, 2 tools (ie, GA and IGT) were combined to capture (1) the time spent by each GP participant, (2) posts made by each GP participant, and (3) the specific activities performed within these sessions to verify GP participants’ active, passive, and nonuse exactly. The platform and the associated analytical capability appear to have several characteristics suitable for both formal and informal CPD activities and may provide an attractive, cost-effective approach to CPD for busy health care professionals, particularly those practicing in rural and regional locations, with the incentive of receiving CPD points.

The novelty of this study centered around the ability to capture the GP participants’ engagement in discussing their own case studies, curated by facilitators, inside a secure and private HVCoP platform for a real-world medical problem (ie, osteoporosis).

The sample of 7 GP participants was small, but it might be speculated from their active and passive behavior that they preferred *practice-based* topics (ie, case studies) rather than didactic information on osteoporosis (ie, topical discussions). An example would be our most engaging case study, case study 5, where GP participants and facilitators discussed an 89-year-old woman who had a history of vertebral fracture noting previous osteoporosis therapy. Previous literature suggests that case studies are important for incentivizing HPs in using Web-based social networks [29,30], especially *practice-based* topics by GPs [13]. This study supports both these areas of previous work; however, given the limitation of our small sample size, this notion still needs to be tested and verified in a future larger study to truly understand and explain this behavior.

The knowledge hub topics that engaged the most GP participants (6 out of 7) were *About Osteoporosis* and *Prevention*. The *About Osteoporosis* topic presented general information about osteoporosis and linked resources for further knowledge acquisition. *Prevention* is knowledge about the prevention of osteoporosis. GPs tend to benchmark their knowledge on specific medical conditions [13,31]. Our study supported this notion as GPs engaged in benchmarking their own knowledge with the *About Osteoporosis* topic. Furthermore, previous research highlights that GPs are more focused on learning about preventive methods for osteoporosis and not necessarily treatment as research in treating osteoporosis continues to address important unanswered questions [32,33]; hence, this may explain the GP participants’ engagement in our *Prevention* topic. Our least active knowledge hub topic concerned shared decision making. This observation was based on the finding that no participants engaged with this topic, which seems to contradict the literature about the notion of patient-physician shared decision making. However, many of those studies looked at the patients’ perception on the matter [34,35], and HPs have expressed doubts about the very notion of shared decision making at its core [34,36]. With our limited sample size, we hypothesized that GP participants may have believed that they were familiar with this topic in their practice and did not need to see/learn more about it from the HVCoP. In addition, GPs might be accustomed to this concept across different medical conditions in their practice already and the intention of joining the CFC Learning Hub was for specific clinical skills and knowledge related to a specific medical condition. Hence, they may have been less willing to devote time to a theme based on overall practice in osteoporosis and communication behavior.

A larger study is required to properly explore the above speculations from our study results. Nevertheless, the results to date strongly suggest that the platform we have developed will be capable of collecting the necessary quantifiable information for analysis in a future larger study and that this platform will prove to be a useful vehicle for health care professionals’ CPD activities.

### Limitations and Future Research

This study has several limitations. As mentioned above, the sample was small. On the contrary, the investigators considered this sample sufficient for the study program to test our measurement and analysis method and GP participants were very active throughout. This study assessed engagement in relation to a single clinical condition (ie, osteoporosis). Different GPs may have an interest in different topics and therefore measuring engagement in relation to other medical topics also needs to be explored in future research to overcome any selection bias associated with sampling in this study. Studies of other HVCoPs indicate that many had an induction session before commencement. GP participants could attend an informal discussion at the study site, both pre- and postparticipation in the program (ie, in a study by Barnett et al [13]). For administrative and logistical reasons, this study did not have such a session which might have encouraged engagement. Instead, we used the first 2 weeks for GP participants to introduce themselves as well as case study 1 and HT 1 to become familiar with the CFC Learning Hub. The study was implemented only in Melbourne, Australia, and the platform might be limited in its application to other contexts (eg, geographical regions with a paucity of experienced specialist advisers or where GPs were uninterested in particular medical conditions). A total of 6 out of the 7 GP participants were females. Hence, we cannot rule out a gender difference in engagement with this CPD tool as designed and tested. A larger sample with a balanced gender ratio should be evaluated in future studies. Furthermore, the analysis was made by 1 researcher following the method described in the Data Collection section. However, data that were gathered in Microsoft Excel were checked with a specialist and the project coordinator for validity. In addition, the approach that we tested to characterize and quantify participation can be automated and this modification should be feasible for future work.

Therefore, although there are some strong insights from our results, further investigation of the perspectives of the GP participants who were involved will also enhance our understanding of their use of the HVCoP. An example would be to compare our results measured here regarding engagement with our pre- and postknowledge testing to triangulate whether our HVCoP for CPD had positive, neutral, or negative knowledge outcomes for GP participants. Another example is qualitatively assessing GP participants in a postuse interview session about their engagement experiences in the CFC Learning Hub. This will be examined in forthcoming publications.

### Conclusions

This study presents a method to quantify GP participants’ hub-related sessions, activities in each session, time spent, and posting behavior as evidence of what potential features can encourage GP participants to engage in learning and knowledge sharing in an HVCoP for CPD. Furthermore, our study suggests new avenues of CPD by providing evidence of learning and knowledge acquisition outside traditional authoritative sources. The study also suggests new ways of tracking the path from engagement to behavioral use in receiving accreditation (ie, CPD points).

Compared with other Web-based educational communities, perhaps our main insight is that GP participants are interested and engaged in practice-based Web-based learning where they can discuss cases with other professionals. This challenges the cost-effectiveness of building large websites with significant libraries of materials to support Web-based learning. Combining the 2 analytical tools made it possible to measure the duration of time spent and the specific activities performed within these sessions to measure the GP participants’ use of the learning hub.

This study can inform a larger study by focusing on creating and facilitating more practice-based topics with GP participants. Furthermore, we propose posting a query topic such as our *Burning Questions* to give an opportunity for GPs to ask general osteoporosis questions not covered in curated case study posts. This query topic can be posted once every 3 to 4 case studies and might help in incentivizing GPs to be engaged with the HVCoP as a means of benchmarking their own current knowledge with the specialists involved.

## Appendix

Multimedia Appendix 1 A brief explanation of Community Fracture Capture Hub design principles from our previous work.

Multimedia Appendix 2 A brief graphical representation of using two analytical tools to process data.

## Abbreviations

CFC: Community Fracture Capture
CPD: continuing professional development
GA: Google Analytics
GP: general practitioner
HP: health care practitioner
HT: hot topics
HVCoP: health virtual community of practice
IGT: igloo statistical tool
OT: other topics
REDCap: Research Electronic Data Capture
VicReN: Victorian Primary Care Practice-Based Research Network
