# Supplementary material for: Quantifying Use of a Health Virtual Community of Practice for General Practitioners’ Continuing Professional Development: A Novel Methodology and Pilot Evaluation
Source: J Med Internet Res. 2019 Nov 27;21(11):e14545. doi: 10.2196/14545 (PMC6906624; doi:10.2196/14545)
Supplement: Multimedia Appendix 1 [file jmir_v21i11e14545_app1.pdf]

Multimedia Appendix 1

Appendix 1. A Brief Explanation of CFC HUB Design Principles from our previous work.

Design Principle	Definition	Example for Implementation
**Rich Profile Information**	A customizable profile for participants enables individual identity, empowerment for social networking opportunities, and a rich presentation of personal content.	Providing a first and last name results in a real identity which increases trust among participants.
**Platform Navigation**	An easy-to-use platform can help in increasing interaction and participation in a HVCoP.	Providing help tips, hints, or a video tutorial in the interface for assisting new and old users in the navigation of the platform.
**Diverse Community**	A diverse non-competing group of health practitioners can increase interaction and participation because of networking opportunities.	Having a mixture of GPs in seniority level (i.e. GP trainees and Senior GPs) sharing their own experiences together.
**Rich Contextual Content**	High quality content provided to participants can be an incentive to increase HVCoP engagement and participation.	Providing resources that are curated by facilitators from verified evidence-based research.
**Patient Information Anonymization**	Having a mechanism to anonymize patient information inside the HVCoP would lead to giving confidence to any health practitioner of protecting privacy of patients.	Any Case Study that would be shared should be filtered and anonymized by facilitators to keep patient privacy secured.
**Human Roles**	Moderators, administrators, and facilitators provide a learning scaffold for participants in a HVCoP.	Moderating online discussions to stop and block inappropriate user actions to maintain the community's safe space and value.
