# Supplementary material for: Quantifying Use of a Health Virtual Community of Practice for General Practitioners’ Continuing Professional Development: A Novel Methodology and Pilot Evaluation
Source: J Med Internet Res. 2019 Nov 27;21(11):e14545. doi: 10.2196/14545 (PMC6906624; doi:10.2196/14545)

Multimedia Appendix 2

Appendix 2. A brief graphical representation of using two analytical tools to process data.

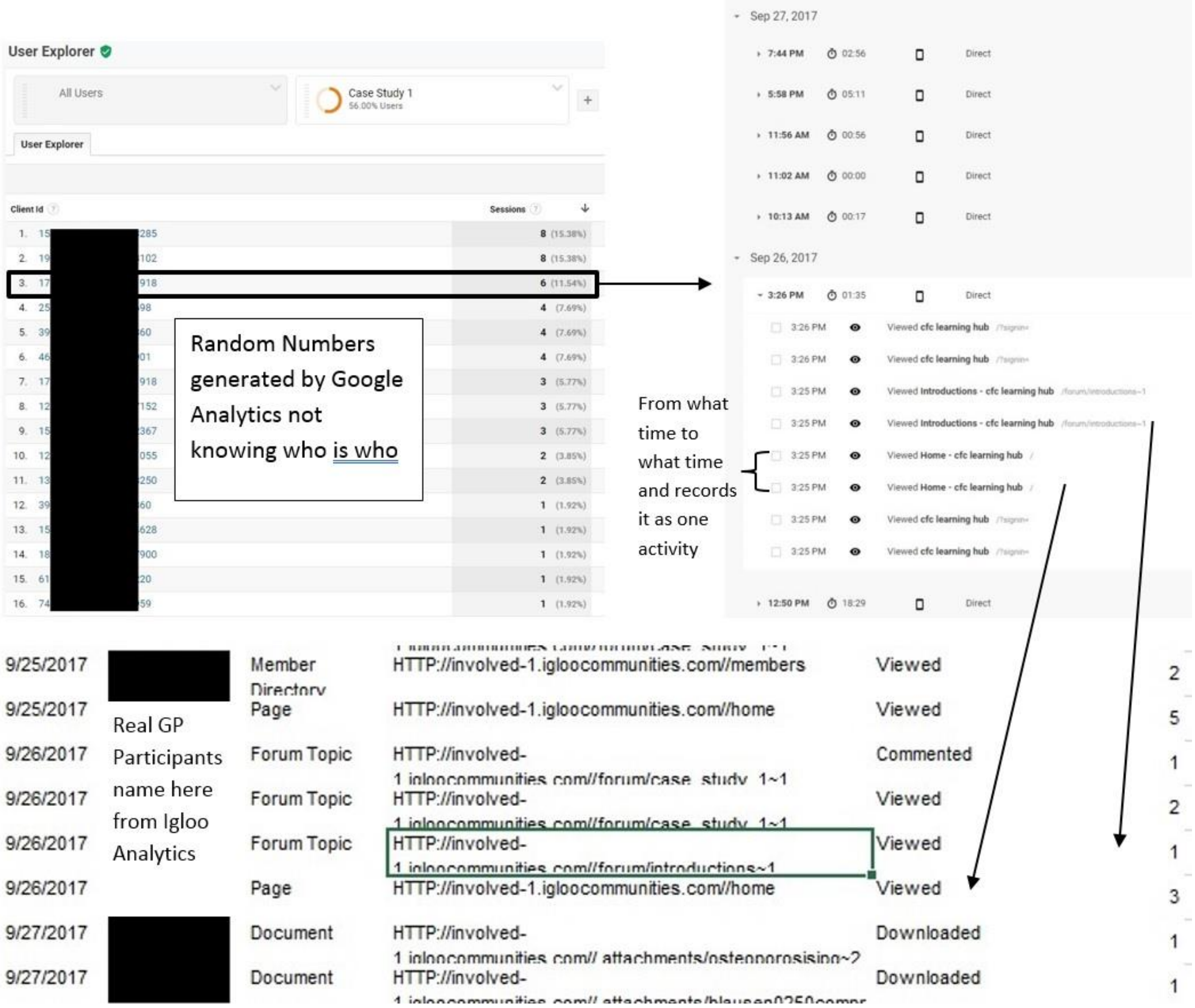

Supplement: Multimedia Appendix 2 [file jmir_v21i11e14545_app2.pdf]
